# Non-synonymous mutations mapped to chromosome X associated with andrological and growth traits in beef cattle

**DOI:** 10.1186/s12864-015-1595-0

**Published:** 2015-05-15

**Authors:** Gregório Miguel Ferreira de Camargo, Laercio R Porto-Neto, Matthew J Kelly, Rowan J Bunch, Sean M McWilliam, Humberto Tonhati, Sigrid A Lehnert, Marina R S Fortes, Stephen S Moore

**Affiliations:** Departamento de Zootecnia, Universidade Estadual Paulista (Unesp), Jaboticabal, SP 14884-900 Brazil; Commonwealth Scientific and Industrial Research Organization, Agriculture Flagship, St Lucia, QLD 4067 Australia; School of Chemistry and Molecular Bioscience, The University of Queensland, St Lucia Brisbane, QLD 4072 Australia; Queensland Alliance for Agriculture and Food Innovation, Centre for Animal Science, The University of Queensland, Brisbane, QLD 4067 Australia

**Keywords:** Non-synonymous SNP, X chromosome, *Bos taurus indicus*, Scrotal circumference, Sperm morphology

## Abstract

**Background:**

Previous genome-wide association analyses identified QTL regions in the X chromosome for percentage of normal sperm and scrotal circumference in Brahman and Tropical Composite cattle. These traits are important to be studied because they are indicators of male fertility and are correlated with female sexual precocity and reproductive longevity. The aim was to investigate candidate genes in these regions and to identify putative causative mutations that influence these traits. In addition, we tested the identified mutations for female fertility and growth traits.

**Results:**

Using a combination of bioinformatics and molecular assay technology, twelve non-synonymous SNPs in eleven genes were genotyped in a cattle population. Three and nine SNPs explained more than 1% of the additive genetic variance for percentage of normal sperm and scrotal circumference, respectively. The SNPs that had a major influence in percentage of normal sperm were mapped to *LOC100138021* and *TAF7L* genes; and in *TEX11* and *AR* genes for scrotal circumference. One SNP in *TEX11* was explained ~13% of the additive genetic variance for scrotal circumference at 12 months. The tested SNP were also associated with weight measurements, but not with female fertility traits.

**Conclusions:**

The strong association of SNPs located in X chromosome genes with male fertility traits validates the QTL. The implicated genes became good candidates to be used for genetic evaluation, without detrimentally influencing female fertility traits.

**Electronic supplementary material:**

The online version of this article (doi:10.1186/s12864-015-1595-0) contains supplementary material, which is available to authorized users.

## Background

In livestock breeding, sires have an important effect in disseminating superior genetic merit, particularly in situations where artificial insemination (AI) is used [1,2]. Sires with better fertility guarantee the efficiency of transmission of the alleles with a superior effect. Andrological parameters are also related to the fertility of sires, which is an important selection trait itself. Sires with good andrological parameters are important, because beef cattle conception rates have economic impact in the production system [3,4]. Improved conception rates increase the economic return. Poor semen quality also impacts on the success rates of reproductive biotechnologies [5].

Andrological traits, such as scrotal circumference measured at 12 months and percentage of morphologically normal sperm measured at 24 months have a moderate and negative genetic correlation with female puberty [6-9] and a moderate and positive genetic correlation with female stayability in the herd [6,8,10,11]. In other words, the selection for higher scrotal circumference and/or higher percentage of normal sperm in young bulls, should lead to female progeny that will be sexually precocious and have a higher probability to stay in the herd. Female fertility traits are of high relevance for beef cattle production in tropical areas. These traits could be from four to thirteen times more important, economically speaking, than carcass and growth traits [12]. Due to cost, andrological traits other than scrotal circumference, are not commonly measured and evaluated in animal breeding programs [13]. The identification of genetic markers associated with the traits could assist in animal breeding, via genomic selection.

Using a GWAS methodology, QTL regions were identified on the bovine X chromosome that potentially influence andrological traits in cattle [14,15]. The aim of this study was to fine-map the QTL regions, focussing on candidate genes to identify possible causative mutations. In future, these variants may be used to construct a low density chip for improved genetic evaluation with a better cost-benefit [16]. Further, customized chips with causative mutations are likely to have a higher transferability among breeds because predictions derived from them do not depend on linkage disequilibrium between the marker assayed and the causal mutation. A GWAS study confirmed that variants in coding regions explain more of the trait variation than random SNPs, exemplifying the important role of missense mutations in genomic evaluation [17].

Candidate genes in the X chromosome QTL regions were chosen according to their biological role. They are: *LOC100138021*, *CENPI*, *TAF7L*, *NXF2*, *CYLC1*, *TEX11*, *AR*, *UXT* and *SPACA5*. The gene *LOC100138021* is a homolog of the *TCP11* gene and plays an important role in spermatogenesis and sperm function in humans [18]; *CENPI* participates in gonadal development and gametogenesis in rats [19]; *TAF7L* could be spermatogenesis-specific and is related to human male infertility [20]; *NXF2*, is an mRNA transporter and its inactivation causes bull infertility [21]; *CYLC1* is a protein of the spermatozoa head with a cytoskeleton function in cattle and humans [22]; *UXT* protein participates in the *AR* transcription regulation in human prostate cells [23] and *SPACA5* codes for protein in the sperm acrosome with lysozyme activity (Gene Ontology). The first four genes are in the QTL associated with percentage of morphologically of normal sperm (39 Mb-59 Mb) and the others in the QTL associated with scrotal circumference (68 Mb-93 Mb) [14,15].

SNPs in *TEX11* and *AR* genes were found to be associated with semen and testis traits in cattle [24]. In this study, the aim was to validate these polymorphisms in another population, so their effect might be confirmed. This validation exercise was extended to include two more candidate genes, not related to the above mentioned QTL: *PLAG1* and *TEKT4*. The *PLAG1* mutation on BTA14 has a pleiotropic effect in many economically important traits in cattle and other species [25,26] and *TEKT4* is a gene associated with spermatozoa motility from a proteomic study in Brahman cattle [27].

A total of eleven genes were chosen as candidates for andrological traits in cattle. The aim was to locate potential causative mutations, defined as non-synonymous SNPs and SNPs or indels in coding and splicing regions, and to verify their association with scrotal circumference (SC) and percentage of normal sperm (PNS) traits in beef bulls. Further, the association of the candidate SNPs with female fertility traits and male growth traits were tested for evaluation of pleiotropic effects.

## Results and discussion

### SNP discovery and genotyping

Based on the 69 bull genomes available, files with SNPs and indels were generated for the target regions. The variants were selected according to their locations (coding regions and splicing sites). For each of seven genes in the SC and PNS QTL regions in chromosome X and for *TEKT4* in chromosome 25, one non-synonymous SNP per gene was selected to be genotyped in the entire population. For *TEX11* two SNPs were tested.

Details about these genes and selected SNPs, such as position and identification number, variation, position in the coding region (CDS) and in the protein and the amino acid change can be found in Table 1. The usual hypothesis applies: changes in the amino acid composition may change protein activity and affect the associated phenotype.

**Table 1:** **Description of candidate genes and interrogated SNP**

**Gene**	Gene position (chromosome - bp)*	**SNP location (chromosome - bp)**	**NCBI SNP number**	**Variation (5’orientation)**	**Position in CDS**	**Position in protein**	**Aminoacid substitution**
*TEKT4*	25: 871,130..877,122	874,677	rs109315777	T/A	584	195	M/K
*LOC100138021*	X:49,737,023..49,737,868	49,737,296	rs461402021	G/C	573	191	I/M
*CENPI*	X:54,969,324..55,038,297	54,971,267	rs134782295	G/A	143	48	S/N
TAF7L**	X:55,127,019..55,144,665	55,133,975	rs445729496	C/T	829/535	277/179	A/T
*NXF2*	X:55,592,336..55,604,610	55,602,546	ss1026566625	T/C	661	221	N/D
*CYLC1*	X:69,903,617..69,933,552	69,914,225	rs477320469	T/A	818	273	Y/F
*UXT*	X:91,468,065..91,474,474	91,472,521	rs132821996	C/T	344	115	S/N
*SPACA5*	X:92,799,368..92,801,705	92,801,539	rs211186307	C/T	109	37	G/S

*position in UMD3.1(Ensembl)

**gene with splicing variants

Using TaqMan assays, 1,021 male cattle were genotyped for twelve SNPs: eight SNPs in the genes described above and four SNP studied in other populations previously. The four SNPs studied before were on the genes *TEX11* (Tex11_r38k and Tex11_r696h), *AR* (AR1_In4) and *PLAG1* (rs109231213) [24,25].

The allelic and genotypic frequencies for all these SNPs are described in Table 2 and 3. All of them presented a good distribution to be used for association analyses. As expected, for the SNPs located in the X chromosome, heterozygotes were not identified in males.

**Table 2:** **Allelic and genotypic frequencies of the SNPs (1,021 bulls)**

**Gene**	**f(C)**	**f(G)**	**f(CC)**	**f(CG)**	**f(GG)**
*PLAG1*	0.64	0.36	0.43	0.42	0.15
*LOC100138021*	0.67	0.33	0.67	0	0.33
	**f(A)**	**f(T)**	**f(AA)**	**f(AT)**	**f(TT)**
*TEKT4*	0.45	0.55	0.22	0.47	0.31
*CYLC1*	0.18	0.82	0.18	0	0.82
	**f(A)**	**f(G)**	**f(AA)**	**f(AG)**	**f(GG)**
*CENPI*	0.40	0.60	0.40	0	0.60
*TEX11_38*	0.32	0.68	0.32	0	0.68
*TEX_696*	0.32	0.68	0.32	0	0.68
*AR*	0.18	0.82	0.18	0	0.82
	**f(C)**	**f(T)**	**f(CC)**	**f(CT)**	**f(TT)**
*NXF2*	0.21	0.79	0.21	0	0.79
*UXT*	0.89	0.11	0.89	0	0.11
*SPACA5*	0.81	0.19	0.81	0	0.19
*TAF7L*	0.68	0.32	0.68	0	0.32

**Table 3:** **Allelic and genotypic frequencies of the SNPs (2,024 cows)**

**Gene**	**f(C)**		**f(G)**		**f(CC)**		**f(CG)**		**f(GG)**	
	**Brahman**	**TC**	**Brahman**	**TC**	**Brahman**	**TC**	**Brahman**	**TC**	**Brahman**	**TC**
*LOC100138021*	0.10	0.78	0.90	0.22	0.01	0.60	0.19	0.36	0.80	0.04
	**f(A)**		**f(T)**		**f(AA)**		**f(AT)**		**f(TT)**	
*TEKT4*	0.82	0.38	0.18	0.62	0.67	0.14	0.31	0.49	0.02	0.37
*CYLC1*	0.47	0.13	0.53	0.87	0.22	0.02	0.49	0.21	0.29	0.77
	**f(A)**		**f(G)**		**f(AA)**		**f(AG)**		**f(GG)**	
*CENPI*	0.92	0.32	0.08	0.68	0.86	0.11	0.12	0.42	0.02	0.47
*TEX11_38*	0.82	0.23	0.18	0.77	0.68	0.05	0.28	0.35	0.04	0.60
*TEX11_696*	0.81	0.22	0.19	0.78	0.67	0.05	0.29	0.35	0.04	0.60
*AR*	0.47	0.15	0.53	0.85	0.21	0.02	0.52	0.26	0.27	0.72
	**f(C)**		**f(T)**		**f(CC)**		**f(CT)**		**f(TT)**	
*NXF2*	0.60	0.14	0.40	0.86	0.36	0.02	0.48	0.21	0.16	0.77
*UXT*	0.59	0.94	0.41	0.06	0.35	0.88	0.48	0.12	0.17	0
*SPACA5*	0.93	0.77	0.07	0.23	0.86	0.59	0.13	0.36	0.01	0.05
*TAF7L*	0.10	0.78	0.90	0.22	0.01	0.60	0.18	0.36	0.81	0.04

### Analysis of linkage disequilibrium

The linkage disequilibrium (LD) was estimated and an arbitrary r^2^ value of 0.80 was considered to indicate that SNPs were in strong linkage disequilibrium. For the bulls (Additional file 1: Table S1), the r^2^ estimates ranged from 0 to 1. However, only two pairs of SNPs had a high estimate of r^2^. The SNPs Tex11_r38k and Tex11_r696h were completely linked (r^2^ value of 1). This means that all animals had the same genotypes for both SNPs and it was impossible to differentiate their effects. For the association analyses, the SNP Tex11_r38k was therefore used to represent both. The SNPs located in *LOC100138021* and *TAF7L* genes also had a high LD (r^2^ value of 0.981); all the other pairs estimates were lower than 0.80. The effect of these SNPs could therefore be analysed separately.

For the cows (Additional file 2: Table S2 and Additional file 3: Table S3), similar results were obtained. The SNPs Tex11_r38k and Tex11_r696h have a high r^2^ value (r^2^ = 0.993 for Brahman cows and r^2^ = 0.927 for TC cows). SNPs located in *LOC100138021* and *TAF7L* genes also had a higher LD (r^2^ value of 0.852 for Brahman cows and 0.827 for TC cows); all the other pairs estimates were lower than 0.80.

Usually, the LD of chromosome X is higher in comparison to the LD of the autosomes [28]; however, the r^2^ values obtained here (r^2^ < 0.80) for most of the SNPs may be explained in terms of population characteristics. The study population consisted of animals of a composite breed and crossbreeds, forming a population where a higher level of recombination will be expected.

### Association analyses

The substitution allelic effect for the named allele of each SNP, standard errors, p-values and the percentage of the additive genetic variance explained were estimated for each studied trait: percentage of normal sperm at 24 months (PNS), scrotal circumference at 12 (SC12), at 18 months (SC18) and at 24 months (SC24) (Table 4). Significant SNPs reported here for SC at three ages and for PNS (Table 4) serve to confirm previously described QTL regions [14,15]. The results show that GWAS research is a very strong tool to find candidate genes. Further, combining the GWAS information with the available genome sequences yield putative causative mutations (non-synonymous and disruptive SNP) associated with SC and PNS.

**Table 4:** **SNP association analysis in bull population (andrological traits)**

**Trait**	**Gene where the SNP is located**	**p-value**	**Allele**	**Effect**	**SE**	**%Va**
PNS	*PLAG1*	0.439	G	0.0079	0.0102	0.24
	*TEKT4*	0.355	A	0.0097	0.0105	0.28
	***LOC100138021***	**0.011**	**G**	**0.0209**	**0.0082**	**2.73**
	***CENPI***	**0.017**	**A**	**0.0180**	**0.0075**	**1.93**
	***TAF7L***	**0.037**	**T**	**0.0173**	**0.0082**	**2.31**
	*NXF2*	0.159	C	0.0120	0.0086	0.82
	*CYLC1*	0.976	A	0.0003	0.0920	0.01
	*TEX11_38*	1.000	A	0.0000	0.0080	0.00
	*AR*	0.217	A	0.0110	0.0088	0.45
	*UXT*	0.313	T	0.0116	0.0114	0.43
	*SPACA5*	0.670	T	0.0035	0.0084	0.03
SC24	*PLAG1*	0.144	G	0.1818	0.1235	0.31
	*TEKT4*	0.307	A	0.1296	0.1263	0.06
	***LOC100138021***	**<0.001**	**G**	**0.5966**	**0.0968**	**2.13**
	*CENPI*	<0.001	A	0.3729	0.0898	0.74
	***TAF7L***	**<0.001**	**T**	**0.6310**	**0.0980**	**2.42**
	*NXF2*	<0.001	C	0.4086	0.1022	0.65
	***CYLC1***	**<0.001**	**A**	**0.7580**	**0.1040**	**2.83**
	***TEX11_38***	**<0.001**	**A**	**0.9929**	**0.0931**	**8.23**
	***AR***	**<0.001**	**A**	**0.8614**	**0.1037**	**4.59**
	***UXT***	**<0.001**	**T**	**0.7374**	**0.1385**	**1.74**
	*SPACA5*	0.088	T	0.1760	0.1024	0.17
SC18	*PLAG1*	0.053	G	0.2583	0.1326	0.62
	*TEKT4*	0.520	A	0.0975	0.1357	0.03
	***LOC100138021***	**<0.001**	**G**	**0.8548**	**0.1025**	**4.12**
	***CENPI***	**<0.001**	**A**	**0.6401**	**0.0953**	**2.32**
	***TAF7L***	**<0.001**	**T**	**0.9212**	**0.1023**	**4.88**
	***NXF2***	**<0.001**	**C**	**0.5537**	**0.1095**	**1.10**
	***CYLC1***	**<0.001**	**A**	**0.8290**	**0.1176**	**2.93**
	***TEX11_38***	**<0.001**	**A**	**1.0550**	**0.0993**	**9.02**
	***AR***	**<0.001**	**A**	**0.8104**	**0.1123**	**3.83**
	***UXT***	**<0.001**	**T**	**0.6638**	**0.1497**	**1.20**
	*SPACA5*	0.004	T	0.3210	0.1096	0.59
SC12	***PLAG1***	**0.035**	**G**	**0.2899**	**0.1363**	**1.13**
	*TEKT4*	0.110	**A**	0.2260	0.1405	0.35
	***LOC100138021***	**<0.001**	**G**	**0.9900**	**0.1041**	**9.23**
	***CENPI***	**<0.001**	**A**	**0.6842**	**0.0977**	**4.35**
	***TAF7L***	**<0.001**	**T**	**1.0460**	**0.1040**	**10.66**
	***NXF2***	**<0.001**	**C**	**0.6755**	**0.1123**	**3.12**
	***CYLC1***	**<0.001**	**A**	**0.8582**	**0.1197**	**4.96**
	***TEX11_38***	**<0.001**	**A**	**1.0540**	**0.1030**	**13.47**
	***AR***	**<0.001**	**A**	**0.8153**	**0.1156**	**5.94**
	***UXT***	**<0.001**	**T**	**0.5491**	**0.1535**	**1.16**
	*SPACA5*	0.062	T	0.2126	0.1130	0.37

Significance (p-value), allelic substitution effect (effect) for the named allele of each SNP, its standard error (SE) and the percentage of additive genetic variance (%Va) explained by the genotypes of each SNP on production of normal sperm at 24 months (PNS), scrotal circumference at 12 months (SC12), at 18 months (SC18) and at 24 months of age (SC24). Significantly associated <0.001 are highlighted in bold. The p-values are uncorrected.

The most significant SNPs associated with PNS (p < 0.05) explained from 1.93% to 2.73% of the additive genetic variance in the bull population. The percentage of additive genetic variance explained by the most significant SNP (p < 0.001) for SC traits varied from 0.65% to 13.47%, in this population. It is worth noticing the effects of SNPs in *AR* and *TEX11* genes for all SC traits and the ones in *LOC10013802* and *TAF7L* for SC12 (Table 4). These percentages of explained genetic variance are considered high for individual mutations. For example, known causative mutations such as those in the calpain and calpastatin genes associated with meat quality explain up to 2% of the phenotypic variance [29]. The very high percentage of additive genetic variance explained by some markers could be explained by the fact that the LD estimates for the X chromosome are higher in comparison to the autosomes. The lower occurrence of cross-overs means that larger DNA fragments are inherited. The SNP associations we observed may therefore report the combined effects of more than one marker. An example of this is the complete linkage of the two SNPs in the *TEX11* gene in the bull population and the inability to differentiate their effects.

The genes *LOC10013802*, *TAF7L*, *CENPI* and *NXF2* were located in the PNS QTL, but they also influence SC traits, indicating a pleiotropic effect for these andrological traits. For these genes and three more (*CYLC1*, *UXT*, *SPACA5*) this study provides the first evidence of an association with male fertility traits in livestock. Polymorphisms in *TAF7L, NXF2* and *LOC10013802* have been associated with male fertility traits in humans and mice, indicating that these genes have conserved roles among mammals [20,30,31], [21,32], [33], respectively. For *CYLC1*, *UXT*, *SPACA5* and *CENPI*, this is the first SNP association study to provide evidence of their influence in male mammal fertility traits.

The SNPs located in *AR* and *TEX11* have been studied before, and their influence on scrotal circumference traits in Brahman and Tropical Composite bulls has been documented [24]. The similar results obtained, in the present study, validate these findings. The SNP in the *AR* gene is located in intron 4 and it is in linkage disequilibrium with important variants located in the promoter region of the gene in cattle. These variants are responsible for the creation/absence of binding sites for *SRY* gene, the gene that initiates sex differentiation in mammals [24]. For *TEX11*, the gene with a SNP that has a large effect on the analysed traits, there is some information based on humans and mouse studies. It is known that this gene acts in gonad development [34], as a meiosis-specific factor [30,35,36] and its loss of function eliminates the spermatocytes. Defects in this gene may cause chromosomal asynapsis and reduction in crossover formation [35]. It has been also shown that it acts in male fertility by competing with estrogen receptor (ERβ) for a specific binding site in the HPIP protein [37]. The non-synonymous SNPs described here changes the amino acid 38 and 696 and the region of *TEX11* protein that binds HPIP protein is from aminoacids 378 to 947 [37], suggesting that the SNP Tex11_r696h may be the best candidate mutation, since it changes an important protein site.

The significant effect (p < 0.005) of the SNP in *PLAG1* for SC12 indicates that this gene also influences scrotal circumference measurements in cattle. This SNP has a pleiotropic effect on a number of growth traits [25] and it was associated with age at 26 cm of SC in cattle [25]. The absence of association of *TEKT4* gene (candidate by a proteomic study with spermatozoa motility in cattle) suggests that there are post-transcriptional changes that might be responsible for affecting the phenotype not related to genotypic variation.

The strong associations seen here confirm that genes located on the X chromosome affect male fertility traits and SNPs in this chromosome should be incorporated in the genetic analysis in order to have better evaluations and genomic values predictions. A recent study validated the importance of coding SNP variants and confirmed that missense SNPs mapped explain the greatest variant for many traits in cattle [17]. The fine-mapping conducted here also highlights the importance to work with putative causative mutation and the benefits that it might bring to the animal breeding and genetics.

The single marker regressions with the top markers fixed are shown in Table 5. The results for PNS and SC traits at different ages indicate that the effects of the top markers are independent in the population. The fixation of the top marker for each trait still allows the significance of other SNPs to be detected. In addition to the LD results shown above, these results indicate that SNPs are segregating separately and that they independently contribute to the traits.

**Table 5:** **Single marker regression fixing the top markers**

**Trait**	**SNP/gene**	**p-value**	**Effect**	**SE**
PNS	*LOC100138021*	0.011	0.0209	0.0082
	*CENPI*	0.008	0.0180	0.0067
SC24	*TEX11_38*	<0.001	0.5526	0.0904
	*AR*	<0.001	0.4176	0.1041
	*TA7L*	<0.001	0.3400	0.0925
SC18	*TEX11_38*	<0.001	0.6256	0.0958
	*TAF7L*	<0.001	0.5708	0.0980
	*AR*	0.003	0.3337	0.1104
SC12	*TEX11_38*	<0.001	0.5226	0.0998
	*TAF7L*	<0.001	0.7073	0.1016
	*AR*	0.006	0.3193	0.1149

Significance (p-value), allelic substitution effect (effect) and its standard error (SE) of each SNP on production of normal sperm at 24 months (PNS), scrotal circumference at 12 months (SC12), at 18 months (SC18) and at 24 months of age (SC24).

The significant SNPs found for these andrological traits are good candidates to be included in customized low density chips for cattle evaluation [16]. Further GWAS and causative mutations studies in the autosomes might be done in the future in order to identify more informative variants for these traits.

These SNPs were also analysed for growth traits in the same males (Table 6). Almost all the SNPs were associated with birth weight and some were also associated with weaning and yearling weights, mainly the SNP in *PLAG1* and *TEX11* genes (p < 0.05) (Table 6). It indicates the selection of the avourable alleles for andrological traits may also select for heavier animals. This result is not surprising given the known genetic correlation between weight and SC [7]. The association of the SNP in *PLAG1* with growth traits confirm previous results reported [25].

**Table 6:** **SNP association analysis in bull population (growth traits)**

**Trait**	**Gene where the SNP is located**	**p-value**	**Allele**	**Effect**	**SE**
BW	*PLAG1*	**<.001**	**G**	**−1.714**	**0.301**
	*TEKT4*	0.786	A	0.084	0.319
	*LOC100138021*	**<.001**	**G**	**−0.847**	**0.234**
	*CENPI*	0.394	A	0.189	0.222
	*TAF7L*	**0.001**	**T**	**0.761**	**0.2355**
	*NXF2*	0.107	C	−0.429	0.264
	*CYLC1*	**<.001**	**A**	**−1.237**	**0.262**
	*TEX11_38*	**<.001**	**A**	**1.586**	**0.223**
	*AR*	**<.001**	**A**	**1.368**	**0.271**
	*UXT*	**<.001**	**T**	**1.391**	**0.363**
	*SPACA5*	**<.001**	**T**	**−1.130**	**0.250**
W200	*PLAG1*	**0.006**	**G**	**−4.151**	**1.490**
	*TEKT4*	0.552	A	−0.907	1.538
	*LOC100138021*	0.153	G	−1.639	1.139
	*CENPI*	0.946	A	−0.068	1.082
	*TAF7L*	0.516	T	0.750	1.161
	*NXF2*	0.807	C	−0.303	1.285
	*CYLC1*	**0.037**	**A**	**−2.759**	**1.307**
	*TEX11_38*	**0.014**	**A**	**2.767**	**1.120**
	*AR*	0.324	A	1.342	1.355
	*UXT*	0.629	T	0.844	1.77
	*SPACA5*	**0.013**	**T**	**−3.033**	**1.21**
W400	*PLAG1*	0.076	G	−3.064	1.712
	*TEKT4*	0.9	A	0.211	1.780
	*LOC100138021*	0.193	G	−1.705	1.3
	*CENPI*	0.742	A	−0.400	1.244
	*TAF7L*	0.515	T	0.858	1.326
	*NXF2*	0.492	C	−1.009	1.474
	*CYLC1*	0.306	A	−1.543	1.503
	*TEX11_38*	**0.047**	**A**	**2.591**	**1.291**
	*AR*	0.205	A	2.016	1.580
	*UXT*	0.829	T	−0.416	2.007
	*SPACA5*	0.068	T	−2.567	1.395
W600	*PLAG1*	0.478	G	−1.490	2.108
	*TEKT4*	0.784	A	−0.575	2.160
	*LOC100138021*	0.164	G	−2.265	1.614
	*CENPI*	0.897	A	−0.187	1.515
	*TAF7L*	0.655	T	0.717	1.629
	*NXF2*	0.464	C	−1.327	1.815
	*CYLC1*	0.218	A	−2.292	1.85
	*TEX11_38*	**0.008**	**A**	**4.186**	**1.571**
	*AR*	0.12	A	2.964	1.888
	*UXT*	0.25	T	2.886	2.496
	*SPACA5*	0.091	T	−2.946	1.725

Significance (p-value), allelic substitution effect (Effect) for the named allele of each SNP and its standard error (SE) of the genotypes of each SNP on birth weight (BW), weight at 200 days (W200), weight at 400 days (W400) and weight at 600 days (W600). Significantly associated <0.05 are highlighted in bold. The p-values are uncorrected.

Overall, there was no association between tested SNP and reproductive traits in females (Table 7). The SNP in the *AR* gene was also associated with the age at the first corpus luteum (AGECL) in Brahman cows (p < 0.05) (Table 7). The selection for this allele may contribute to later cycling cows.

**Table 7:** **SNP association analysis in cow population**

			**Brahman**			**Tropical Composite**		
**Trait**	**Gene**	**Allele**	**p-value**	**Effect**	**SE**	**p-value**	**Effect**	**SE**
AGECL	*TEKT4*	A	0.074	−14.14	7.845	0.732	−1.78	5.323
	*LOC100138021*	G	0.382	8.489	9.701	0.588	−3.643	6.792
	*CENPI*	A	0.462	−7.269	9.916	0.556	3.661	6.271
	*TAF7L*	T	0.272	−10.66	9.661	0.498	4.602	6.817
	*NXF2*	C	0.373	−6.142	6.883	0.86	1.362	8.079
	*CYLC1*	A	0.861	1.122	6.713	0.29	8.478	7.972
	*TEX11_38*	A	0.932	0.6909	8.611	0.775	1.891	6.825
	*TEX11_696*	A	0.821	1.859	8.549	0.805	1.661	6.987
	*AR*	**A**	**0.022**	**15.79**	**6.833**	0.747	−2.314	7.377
	*UXT*	T	0.062	13.27	7.039	0.918	−1.027	10.59
	*SPACA5*	T	0.196	18.18	13.95	0.874	−1.091	7.238
PPAI	*TEKT4*	A	0.276	−9.85	8.994	0.792	1.287	5.048
	*LOC100138021*	G	0.138	15.61	10.43	0.579	3.636	6.607
	*CENPI*	A	0.574	5.844	10.48	0.281	6.447	5.943
	*TAF7L*	T	0.224	13.01	10.62	0.467	4.797	6.604
	*NXF2*	C	0.907	−0.8449	7.67	0.331	7.511	7.703
	*CYLC1*	A	0.616	−3.604	7.283	0.369	6.873	7.629
	*TEX11_38*	A	0.468	−6.915	9.559	0.278	7.073	6.490
	*TEX11_696*	A	0.593	−5.026	9.504	0.246	7.696	6.598
	*AR*	A	0.665	3.195	7.511	0.485	4.831	6.943
	*UXT*	T	0.316	−7.496	7.436	0.516	−6.693	10.37
	*SPACA5*	T	0.19	18.91	14.31	0.823	1.479	6.849

Significance (p-value), allelic substitution effect (Effect) for the named allele of each SNP and its standard error (SE) of the genotypes of each SNP on age at first corpus luteum (AGECL) and postpartum anestrus interval (PPAI).

The TaqMan assays were also used to genotype 90 Angus cattle in order to verify the origin of the alleles (Table 8). For the SNPs located in the genes *LOC100138021*, *TEX11*, *AR*, *NXF2*, *UXT* and *TAF7L*, one of the alleles is fixed in the Angus population and for the genes *CYLC1*, *CENPI* and *PLAG1*, one allele is close to fixation. Fortes et al. found similar results for the *PLAG1* SNP [25]. The Brahman population of the study represented all genotypes for the SNPs. The source of variation for ten out of twelve of the genes studied therefore appears to be the zebu cattle.

**Table 8:** **Allelic frequencies in Angus population (90 animals)**

**Gene**	**f(C)**	**f(G)**
*PLAG1*	0.94	0.06
*LOC100138021*	1	0
	**f(A)**	**f(T)**
*TEKT4*	0.18	0.82
*CYLC1*	0.01	0.99
	**f(A)**	**f(G)**
*CENPI*	0.06	0.94
*TEX11_38*	0	1
*TEX11_696*	0	1
*AR*	0	1
	**f(C)**	**f(T)**
*NXF2*	0	1
*UXT*	1	0
*SPACA5*	0.77	0.23
*TAF7L*	1	0

## Conclusions

The QTL on chromosome X associated with bull fertility have been confirmed in an independent population. Putative causative mutations in the X chromosome influence the production of normal sperm and scrotal circumference of young bulls in Zebu cattle and their crossbreds. They are good candidate SNPs to be incorporated in low-density chips that could facilitate genetic evaluation. Moreover, the information provided on key genes may serve as basis for further functional experiments. Pleiotropic effects across andrological and growth traits were reported; nevertheless these mutations had no impact on female fertility traits.

## Methods

### Animals and phenotypic data

Animal Care and Use Committee approval was not required for this study because the data were obtained from existing phenotypic databases and DNA storage banks as described below.

Data from 1,021 bulls whose breeds were Brahman (n = 113), Tropical Composite (n = 741) and crossbreeds (n = 167) from five farms born from 2004 to 2009 were used in the current study. These animals were bred by the Beef CRC and the experimental design as well as the general population description of the CRC were reported previously [7,9]. Importantly, the animals used in this study had not been genotyped for any of the previous CRC studies.

The traits utilized in this study were: scrotal circumference at 12 (SC12), at 18 months (SC18) and at 24 months (SC24) and percentage of normal sperm at 24 months (PNS), birth weight (BW), weight at 200 days (W200), weight at 400 days (W400) and weight at 600 days (W600). All the traits were measured in the same bulls. Details about the measurements of the andrological traits can be found in [24].

Data from 935 Brahman cows and 1,089 Tropical Composite cows also from Beef CRC population were used in the current study. The traits analyzed were: age at first corpus luteum (AGECL) and postpartum anestrus interval (PPAI). More information about the population, breeds and the phenotypes could be found in [38].

In order to determine the proportion of *Bos taurus* alleles, 90 Angus cattle were also genotyped using the same methodology described below and the frequencies were compared.

### Bioinformatic analyses

The genome of 64 bulls (from CSIRO Animal, Food and Health Sciences in St Lucia, Brisbane, QLD, Australia) was used to generate a VCF (variant call format) files with variants (SNPs and indels) information for the target regions using the software SNVer version 0.4.1.[39] The breed of the 64 bulls are: 42 Brahman, 14 Hereford and 8 Senepol.

Variant Effect Predictor (VEP) is an online tool from Ensembl website (http://www.ensembl.org/info/docs/tools/vep/index.html) was used to predict the functional consequences of detected variants. The aim was to find disruptive variants with a major effect on the traits. So, we started looking for non-synonymous SNPs and SNPs/indels in coding regions and splicing sites of the candidate genes listed above.

### Genotyping of selected SNPs

Custom TaqMan assays were developed for the novel selected SNPs according to TaqMan Array Design Tool [40] and are listed in Additional file 4: Table S4. SNPs in *TEX11* (Tex11_r38k and Tex11_r696h), *AR* (AR1_In4) and *PLAG1 (*rs109231213) genes, primers and probes were used as described by [24] and [26], respectively.

### Analysis of linkage disequilibrium

The linkage disequilibrium (r^2^) was estimated using the Plink program (http://pngu.mgh.harvard.edu/~purcell/plink/, accessed 5 June 2014) to determine which SNPs were more frequently inherited together. Considering two loci with two alleles for each locus (A/a and B/b), the following formula was used:

*r*^2^ = [ *f*(*AB*) *x f*(*ab*) – *f*(*Ab*) *x f*(*aB*)]^2^/ *f*(*A*) *x f*(*a*) *x f*(*B*) *x f*(*b*)] = *D*^2^/ [*f*(*A*) *x f*(*a*) *x f*(*B*) *x f*(*b*)] where ‘f’ is the frequency and D = f(AB) - f(A) x f(B)

### Statistical analyses

The single marker regression was examined for genotyped animals using a mixed model analysis of variance with ASREML software. The mixed model is described below:

$$ {y}_i = X\beta + Z\mu + {S}_j{a}_j + {e}_i $$

Where *yi* represents the phenotypic measurement for the *i*^th^ animal, X is the incidence matrix relating fixed effects in β with observations in *y*, *Z* is the incidence matrix relating to random additive polygenic effects of animal in μ with observations in *y* and *S*_*j*_ is the observed animal genotype for the j^th^ SNP (coded as 0, 1 or 2 to represent the number of copies of the B allele), if the SNPs were located in the X chromosome and males were genotyped, they were coded as 0 or 2 (since there is no heterozygous), *a*_j_ is the estimated SNP effect, lastly *e*_*i*_ is the random residual effect. For SC12, SC18, SC24 and PNS, the same fixed effects were used for each trait. These fixed effects included contemporary group (animals born in the same year and raised together), the interaction of year and month of birth and breed. For AGECL and PPAI, the fixed effects were contemporary group (i.e., group of heifers born in the same year and raised together), herd of origin and age of dam. For bull growth traits, the fixed effects included cohort origin and age. The p-values were not corrected for multiple testing.

The percentage of the genetic variance accounted by the j^th^ SNP was estimated according to the formula $\%Vj=100.\frac{2{p}_j{q}_j{a}_j^2}{\sigma_g^2}$ where p and q are the allele frequencies for the j^th^ SNP estimated across the entire population, a_j_ is the estimated additive effect of the j^th^ SNP on the trait under analysis, and σ_*g*_^2^ is the REML estimate of the (poly-) genetic variance for the trait.

The single marker regression was also done fixing the top marker (higher F statistic) for each trait. The p-values of the other markers were recalculated consecutively until no marker has a significant p-value. The aim of these analyses is to verify the independence of the effect among the markers.

## Availability of supporting data

Animal genotypes for all markers are available as Additional file 5: Table S5.

## Additional files

Additional file 1: Table S1. Estimated pairwise r^2^ values for the SNPs studied in bulls. Additional file 2: Table S2. Estimated pairwise r^2^ values for the SNPs studied in Brahman cows. Additional file 3: Table S3. Estimated pairwise r^2^ values for the SNPs studied in Tropical Composite cows. Additional file 4: Table S4. SNPs genotyped and nucleotide sequences of primers and probes used in TaqMan® Assays. Additional file 5: Table S5. Animal genotypes for all markers.

## References

[1] Dahlen C, Larson J, Lamb GC (2014). Impacts of Reproductive Technologies on Beef Production in the United States. Adv Exp Med Biol.

[2] Kues WA, Rath D, Niemann H (2008). Reproductive biotechnology in farm animals goes genomics. CAB Reviews: Perspectives in Agriculture, Veterinary Science, Nutrition and Natural Resources.

[3] Samarajeewa S, Hailu G, Jeffrey SR, Bredahl M (2012). Analysis of production efficiency of beef cow/calf farms in Alberta. Appl Econ.

[4] Wolfova M, Wolf J, Zahradkova R, Pribyl J, Dano J, Krupa E (2005). Breeding objectives for beef cattle used in different production systems - 2. Model application to production systems with the Charolais breed. Livest Prod Sci.

[5] Brumatti RC, Ferraz JBS, Eler JP (2013). Influence of mating and reproductive techniques in selection programs of beef cattle and its impact on the cost and production of young bulls. Livest Res Rural Dev.

[6] Johnston DJ, Corbet NJ, Barwick SA, Wolcott ML, Holroyd RG (2014). Genetic correlations of young bull reproductive traits and heifer puberty traits with female reproductive performance in two tropical beef genotypes in northern Australia. Anim Prod Sci.

[7] Corbet NJ, Burns BM, Johnston DJ, Wolcott ML, Corbet DH, Venus BK (2013). Male traits and herd reproductive capability in tropical beef cattle. 2. Genetic parameters of bull traits. Anim Prod Sci.

[8] Santana ML, Eler JP, Ferraz JBS, Mattos EC (2012). Genetic relationship between growth and reproductive traits in Nellore cattle. Animal.

[9] Burns BM, Gazzola C, Holroyd RG, Crisp J, McGowan MR (2011). Male Reproductive Traits and Their Relationship to Reproductive Traits in Their Female Progeny: A Systematic Review. Reprod Domest Anim.

[10] Buzanskas ME, Grossi DA, Baldi F, Barrozo D, Silva LOC, Torres RAA (2010). Genetic associations between stayability and reproductive and growth traits in Canchim beef cattle. Livest Sci.

[11] Van Melis MH, Eler JP, Rosa GJM, Ferraz JBS, Figueiredo LGG, Mattos EC (2010). Additive genetic relationships between scrotal circumference, heifer pregnancy, and stayability in Nellore cattle. J Anim Sci.

[12] Brumatti RC, Ferraz JBS, Eler JP, Formigonni IB (2011). DEVELOPMENT OF SELECTION INDEX IN BEEF CATTLE UNDER THE FOCUS OF A BIO-ECONOMIC MODEL. Archivos de Zootecnia.

[13] Silva MR, Pedrosa VB, Silva JBC, Herrera LGG, Eler JP, Albuquerque LG (2012). Genetic parameters of andrological traits in bovine. Archivos De Medicina Veterinaria.

[14] Fortes MRS, Reverter A, Hawken RJ, Bolormaa S, Lehnert SA (2012). Candidate Genes Associated with Testicular Development, Sperm Quality, and Hormone Levels of Inhibin, Luteinizing Hormone, and Insulin-Like Growth Factor 1 in Brahman Bulls. Biol Reprod.

[15] Fortes MRS, Reverter A, Kelly M, McCulloch R, Lehnert SA (2013). Genome-wide association study for inhibin, luteinizing hormone, insulin-like growth factor 1, testicular size and semen traits in bovine species. Andrology.

[16] Snelling WM, Cushman RA, Fortes MRS, Reverter A, Bennett GL, Keele JW (2012). PHYSIOLOGY AND ENDOCRINOLOGY SYMPOSIUM: How single nucleotide polymorphism chips will advance our knowledge of factors controlling puberty and aid in selecting replacement beef females. J Anim Sci.

[17] Koufariotis L, Chen Y-PP, Bolormaa S, Hayes BJ (2014). Regulatory and coding genome regions are enriched for trait associated variants in dairy and beef cattle. BMC Genomics.

[18] Liu Y, Jiang M, Li C, Yang P, Sun H, Tao D (2011). Human t-Complex Protein 11 (TCP11), a Testis-Specific Gene Product, Is a Potential Determinant of the Sperm Morphology. Tohoku J Exp Med.

[19] Roberts RG, Kendall E, Vetrie D, Bobrow M (1996). Sequence and chromosomal location of a human homologue of LRPR1, an FSH primary response gene. Genomics.

[20] Stouffs K, Willems A, Lissens W, Tournaye H, Van Steirteghem A, Liebaers I (2006). The role of the testis-specific gene hTAF7L in the aetiology of male infertility. Mol Hum Reprod.

[21] Pan Q, Ju Z, Huang J, Zhang Y, Qi C, Gao Q, Zhou L, Li Q, Wang L, Zhong J et al. PLCz Functional Haplotypes Modulating Promoter Transcriptional Activity Are Associated with Semen Quality Traits in Chinese Holstein Bulls. Plos One 2013, 8(3):e58795. 10.1371/journal.pone.0058795 PMC3598912 23554927

[22] Hess H, Heid H, Franke WW (1993). MOLECULAR CHARACTERIZATION OF MAMMALIAN CYLICIN, A BASIC-PROTEIN OF THE SPERM HEAD CYTOSKELETON. J Cell Biol.

[23] Markus SM, Taneja SS, Logan SK, Li WH, Ha S, Hittelman AB (2002). Identification and characterization of ART-27, a novel coactivator for the androgen receptor N terminus. Mol Biol Cell.

[24] Lyons RE, Loan NT, Dierens L, Fortes MRS, Kelly M, McWilliam SS (2014). Evidence for positive selection of taurine genes within a QTL region on chromosome X associated with testicular size in Australian Brahman cattle. BMC Genet.

[25] Fortes MRS, Kemper K, Sasazaki S, Reverter A, Pryce JE, Barendse W (2013). Evidence for pleiotropism and recent selection in the PLAG1 region in Australian Beef cattle. Anim Genet.

[26] Karim L, Takeda H, Lin L, Druet T, Arias JAC, Baurain D (2011). Variants modulating the expression of a chromosome domain encompassing PLAG1 influence bovine stature. Nat Genet.

[27] Thepparat T, Katawatin S, Vongpralub T, Duangjinda M, Thammasirirak S, Utha A (2012). Separation of bovine spermatozoa proteins using 2D-PAGE revealed the relationship between tektin-4 expression patterns and spermatozoa motility. Theriogenology.

[28] Porto-Neto LR, Kijas JW, Reverter A (2014). The extent of linkage disequilibrium in beef cattle breeds using high-density SNP genotypes. Genet Sel Evol.

[29] Barendse W, Harrison BE, Hawken RJ, Ferguson DM, Thompson JM, Thomas MB (2007). Epistasis between calpain 1 and its inhibitor calpastatin within breeds of cattle. Genetics.

[30] Zheng K, Yang F, Wang PJ (2010). Regulation of Male Fertility by X-Linked Genes. J Androl.

[31] Zhou H, Grubisic I, Zheng K, He Y, Wang PJ, Kaplan T (2013). Taf7l cooperates with Trf2 to regulate spermiogenesis. Proc Natl Acad Sci U S A.

[32] Zhou J, McCarrey JR, Wang PJ. A 1.1-Mb Segmental Deletion on the X Chromosome Causes Meiotic Failure in Male Mice. Biol Reprod 2013, 88(6):159. 10.1095/biolreprod.112.106963 PMC4070866 23677977

[33] Safronova LD, Kudryavtsev IV, Kudryavtsev PI (2002). Sterility of males determined by functional features of the mouse spermatozoa bearing t-complex. Ontogenez.

[34] Houmard B, Small C, Yang LZ, Naluai-Cecchini T, Cheng E, Hassold T (2009). Global Gene Expression in the Human Fetal Testis and Ovary. Biol Reprod.

[35] Yang F, Gell K, Van der Heijden GW, Eckardt S, Leu NA, Page DC (2008). Meiotic failure in male mice lacking an X-linked factor. Genes Dev.

[36] Adelman CA, Petrini JHJ (2008). ZIP4H (TEX11) deficiency in the mouse impairs meiotic double strand break repair and the regulation of crossing over. PLoS Genet.

[37] Yu YH, Siao FP, Hsu LCL, Yen PH (2012). TEX11 Modulates Germ Cell Proliferation by Competing with Estrogen Receptor beta for the Binding to HPIP. Mol Endocrinol.

[38] Fortes MRS, Reverter A, Nagaraj SH, Zhang Y, Jonsson NN, Barris W (2011). A single nucleotide polymorphism-derived regulatory gene network underlying puberty in 2 tropical breeds of beef cattle. J Anim Sci.

[39] Wei Z, Wang W, Hu P, Lyon GJ, Hakonarson H (2011). SNVer: a statistical tool for variant calling in analysis of pooled or individual next-generation sequencing data. Nucleic Acids Res.

[40] Custom TaqMan Assays using the Custom TaqMan® Assay Design Tool on the Applied Biosystems, 2010

