# Supplementary material for: Non-synonymous mutations mapped to chromosome X associated with andrological and growth traits in beef cattle
Source: BMC Genomics. 2015 May 15;16(1):384. doi: 10.1186/s12864-015-1595-0 (PMC4432507; doi:10.1186/s12864-015-1595-0)
Supplement: Additional file 1: Table S1. — Estimated pairwise r2 values for the SNPs studied in bulls. [file 12864_2015_1595_MOESM1_ESM.doc]

**Table S1:** Estimated pairwise r^2^ values^*^ for the SNPs studied in bulls.

| **SNPs position** | **14: 25,219,343**  **(*PLAG1*)** | **25:**  **874,677**  **(*TEKT4*)** | **X:**  **49,737,296**  **(*LOC100138021)*** | **X: 54,971,267 (*CENPI)*** | **X: 55,133,073 (*TAF7L)*** | **X: 55,602,546 (*NXF2)*** | **X: 69,914,225 (*CYLC1)*** | **X:85,042,933 (*TEX11_38*)** | **X: 85,042,933 (*TEX11_696*)** | **X: 88,418,702 (*AR*)** | **X: 91,472,521 (*UXT)*** | **X: 92,801,539 (*SPACA5)*** |
| --- | --- | --- | --- | --- | --- | --- | --- | --- | --- | --- | --- | --- |
| **14: 25,219,343 (*PLAG1*)** | **-** | 0 | 0 | 0 | 0 | 0 | 0 | 0 | 0 | 0 | 0 | 0.001 |
| **25:874,677 (*TEKT4*)** |  | **-** | 0.021 | 0.011 | 0.020 | 0.015 | 0.010 | 0.016 | 0.016 | 0.003 | 0.016 | 0.002 |
| **X: 49,737,296 (*LOC100138021)*** |  |  | **-** | 0.673 | **0.981** | 0.556 | 0.2 | 0.242 | 0.252 | 0.071 | 0.141 | 0.012 |
| **X: 54,971,267 (*CENPI)*** |  |  |  | **-** | 0.692 | 0.389 | 0.13 | 0.14 | 0.151 | 0.043 | 0.09 | 0.015 |
| **X: 55,133,073 (*TAF7L)*** |  |  |  |  | **-** | 0.569 | 0.198 | 0.253 | 0.264 | 0.084 | 0.144 | 0.010 |
| **X: 55,602,546 (*NXF2)*** |  |  |  |  |  | **-** | 0.076 | 0.112 | 0.117 | 0.029 | 0.114 | 0 |
| **X: 69,914,225 (*CYLC1)*** |  |  |  |  |  |  | **-** | 0.394 | 0.4 | 0.061 | 0.086 | 0.034 |
| **X: 85,042,933 (*TEX11_38)*** |  |  |  |  |  |  |  | **-** | **1.0** | 0.341 | 0.21 | 0.094 |
| **X: 85,042,933 (*TEX11_696*)** |  |  |  |  |  |  |  |  | - | 0.342 | 0.216 | 0.094 |
| **X: 88,418,702**  **(*AR*)** |  |  |  |  |  |  |  |  |  | **-** | 0.187 | 0.016 |
| **X: 91,472,521 (*UXT*)** |  |  |  |  |  |  |  |  |  |  | **-** | 0.023 |

^*^The r^2^ presented was the squared correlations between the coded SNPs.
