# Supplementary material for: Non-synonymous mutations mapped to chromosome X associated with andrological and growth traits in beef cattle
Source: BMC Genomics. 2015 May 15;16(1):384. doi: 10.1186/s12864-015-1595-0 (PMC4432507; doi:10.1186/s12864-015-1595-0)
Supplement: Additional file 2: Table S2. — Estimated pairwise r2 values for the SNPs studied in Brahman cows. [file 12864_2015_1595_MOESM2_ESM.doc]

**Table S2: Estimated pairwise r^2^ values^*^ for the SNPs studied in Brahman cows.**

| **SNPs position** | **25:**  **874,677**  **(*TEKT4*)** | **X:**  **49,737,296**  **(*LOC100138021)*** | **X: 54,971,267 (*CENPI)*** | **X: 55,133,073 (*TAF7L)*** | **X: 55,602,546 (*NXF2)*** | **X: 69,914,225 (*CYLC1)*** | **X: 85,042,933 (*TEX11_38*)** | **X: 85,178,633 (*TEX11_696*)** | **X: 88,418,702 (*AR*)** | **X: 91,472,521 (*UXT)*** | **X: 92,801,539 (*SPACA5)*** |
| --- | --- | --- | --- | --- | --- | --- | --- | --- | --- | --- | --- |
| **25:874,677 (*TEKT4*)** | - | 0.003 | 0.001 | 0.001 | 0.001 | 0.001 | 0.0012 | 0.0012 | 0.002 | 0.001 | 0 |
| **X: 49,737,296 (*LOC100138021)*** |  | - | 0.555 | **0.852** | 0.138 | 0.029 | 0.068 | 0.070 | 0.008 | 0.007 | 0.004 |
| **X: 54,971,267 (*CENPI)*** |  |  | - | 0.567 | 0.064 | 0.018 | 0.043 | 0.043 | 0.006 | 0.006 | 0 |
| **X: 55,133,073 (*TAF7L)*** |  |  |  | - | 0.130 | 0.036 | 0.075 | 0.073 | 0.013 | 0.011 | 0.006 |
| **X: 55,602,546 (*NXF2)*** |  |  |  |  | - | 0.003 | 0 | 0 | 0.004 | 0.020 | 0.015 |
| **X: 69,914,225 (*CYLC1)*** |  |  |  |  |  | - | 0.139 | 0.140 | 0.016 | 0.009 | 0.048 |
| **X: 85,042,933 (*TEX11_38)*** |  |  |  |  |  |  | - | **0.993** | 0.173 | 0.087 | 0.3 |
| **X: 85,178,633 (*TEX11_696)*** |  |  |  |  |  |  |  | - | 0.178 | 0.086 | 0.289 |
| **X: 88,418,702**  **(*AR*)** |  |  |  |  |  |  |  |  | - | 0.133 | 0.067 |
| **X: 91,472,521 (*UXT*)** |  |  |  |  |  |  |  |  |  | - | 0.057 |

^*^The r^2^ presented was the squared correlations between the coded SNPs.
