# Supplementary material for: Non-synonymous mutations mapped to chromosome X associated with andrological and growth traits in beef cattle
Source: BMC Genomics. 2015 May 15;16(1):384. doi: 10.1186/s12864-015-1595-0 (PMC4432507; doi:10.1186/s12864-015-1595-0)
Supplement: Additional file 3: Table S3. — Estimated pairwise r2 values for the SNPs studied in Tropical Composite cows. [file 12864_2015_1595_MOESM3_ESM.doc]

**Table S3: Estimated pairwise r^2^ values^*^ for the SNPs studied in Tropical Composite cows.**

| **SNPs position** | **25:**  **874,677**  **(*TEKT4*)** | **X:**  **49,737,296**  **(*LOC100138021)*** | **X: 54,971,267 (*CENPI)*** | **X: 55,133,073 (*TAF7L)*** | **X: 55,602,546 (*NXF2)*** | **X: 69,914,225 (*CYLC1)*** | **X: 85,042,933 (*TEX11_38*)** | **X: 85,178,633 (*TEX11_696*)** | **X: 88,418,702 (*AR*)** | **X: 91,472,521 (*UXT)*** | **X: 92,801,539 (*SPACA5)*** |
| --- | --- | --- | --- | --- | --- | --- | --- | --- | --- | --- | --- |
| **25:874,677 (*TEKT4*)** | - | 0 | 0.002 | 0 | 0 | 0 | 0.004 | 0.004 | 0.001 | 0.001 | 0 |
| **X: 49,737,296 (*LOC100138021)*** |  | - | 0.523 | **0.827** | 0.491 | 0.174 | 0.189 | 0.186 | 0.044 | 0.086 | 0.024 |
| **X: 54,971,267 (*CENPI)*** |  |  | - | 0.546 | 0.294 | 0.116 | 0.124 | 0.106 | 0.018 | 0.042 | 0.035 |
| **X: 55,133,073 (*TAF7L)*** |  |  |  | - | 0.481 | 0.241 | 0.179 | 0.184 | 0.038 | 0.066 | 0.019 |
| **X: 55,602,546 (*NXF2)*** |  |  |  |  | - | 0.041 | 0.078 | 0.080 | 0.023 | 0.034 | 0.002 |
| **X: 69,914,225 (*CYLC1)*** |  |  |  |  |  | - | 0.333 | 0.353 | 0.033 | 0.040 | 0.035 |
| **X: 85,042,933 (*TEX11_38)*** |  |  |  |  |  |  | - | **0.927** | 0.278 | 0.156 | 0.077 |
| **X: 85,178,633 (*TEX11_696)*** |  |  |  |  |  |  |  | - | 0.316 | 0.167 | 0.078 |
| **X: 88,418,702**  **(*AR*)** |  |  |  |  |  |  |  |  | - | 0.200 | 0.006 |
| **X: 91,472,521 (*UXT*)** |  |  |  |  |  |  |  |  |  | - | 0.010 |

^*^The r^2^ presented was the squared correlations between the coded SNPs.
