# Supplementary material for: Non-synonymous mutations mapped to chromosome X associated with andrological and growth traits in beef cattle
Source: BMC Genomics. 2015 May 15;16(1):384. doi: 10.1186/s12864-015-1595-0 (PMC4432507; doi:10.1186/s12864-015-1595-0)
Supplement: Additional file 4: Table S4. — SNPs genotyped and nucleotide sequences of primers and probes used in TaqMan® Assays. [file 12864_2015_1595_MOESM4_ESM.doc]

**Table S4:** SNPs genotyped and nucleotide sequences of primers and probes used in TaqMan® Assays.

| SNP number | Primer (5' – 3') | Probe (5' – 3') |
| --- | --- | --- |
| [rs109315777](http://www.ncbi.nlm.nih.gov/projects/SNP/snp_ref.cgi?rs=109315777) | ACCCAGACCCACCGAATCT  CGAGCTCATCCGGAACATTCAG | VIC- ACGGCCTGCTTGATG  FAM- CGGCCTGCATGATG |
| [rs461402021](http://www.ncbi.nlm.nih.gov/projects/SNP/snp_ref.cgi?rs=461402021) | TTCTGGTGACCATCGTAGCTTTC  GGGTGATGATACAGTCCTGTTTTGG | VIC- CAGTTTCTCCAAGATTAGT  FAM- CAGTTTCTCCAACATTAGT |
| [rs134782295](http://www.ncbi.nlm.nih.gov/projects/SNP/snp_ref.cgi?rs=134782295) | GGACCTAAATGACTCAAAGAGCATCT  GCTTGATCATCAGCGCCTTCATA | VIC- TGTGGACAGAACAGTACTGT  FAM- TGGACAGAACAATACTGT |
| rs445729496 | TGAGGTGGAACAAACTACACAGAAA  GGAAGTGAAAAGACTGCTGTGTTC | VIC- TGAAGCTGTCAATGCCCGTA  FAM- TGAAGCTGTCAATACCCGTA |
| ss1026566625 | CCACACCCACCCTCAAATTACTAC  CAATCTCTTGACCTCCAGAAGCT | VIC- TGTCAGCCATACTTGGGTCA  FAM- CAGCCATACCTGGGTCA |
| rs483088766 | TGCTTCCTAAATCGTGCACTTGA  TCCCACTGTACCTGTTGTTTTCAG | VIC- TCTGATCCGTAAATGC  FAM- TCTGATCCATAAATGC |
| [rs477320469](http://www.ncbi.nlm.nih.gov/projects/SNP/snp_ref.cgi?rs=477320469) | TTTTTGCATCCTTCTTTGTTGGCTT  GGATGCTGAATCCATGGAATTTGAT | VIC- ATTCTGTGAATAATTCTT  FAM- TCTGTGAAAAATTCTT |
| [rs132821996](http://www.ncbi.nlm.nih.gov/projects/SNP/snp_ref.cgi?rs=132821996) | CCTCATGCAATGGACTTACTCTGT  GGCAGAAGCTCTCAAGTTCATTG | VIC- TCGTAAGAGCAGTCTCCT  FAM- CGTAAGAGCAATCTCCT |
| [rs211186307](http://www.ncbi.nlm.nih.gov/projects/SNP/snp_ref.cgi?rs=211186307) | GGTTCTCACAGTCTCCAATGGTAT  TGGCAAAGAAGCTGGAAGCA | VIC- CCTCAACGGCTTCAAG  FAM- CCTCAACAGCTTCAAG |
